# Supplementary material for: Sweeteners’ Influence on In Vitro α-Glucosidase Inhibitory Activity, Cytotoxicity, Stability and In Vivo Bioavailability of the Anthocyanins from Lingonberry Jams
Source: Foods. 2023 Jun 30;12(13):2569. doi: 10.3390/foods12132569 (PMC10340494; doi:10.3390/foods12132569)
Supplement: Supplementary file 1 [file foods-12-02569-s001.zip › foods-2425713-supplementary.pdf]

Impact of different sweeteners on *in vitro* α-glucosidase inhibitory activity, cytotoxicity of lingonberry jams and on stability and *in vivo* bioavailability of their anthocyanins

Teodora Scrob [1,2], Gabriela Adriana Filip [3], Ioana Baldea [3], Sânziana Maria Varodi [1] and Claudia Cimpoiu [1,2,*]

Table S1. The Student's *t*-test results (*t*-values) for statistical comparison of individual anthocyanins after different intervals of storage at 4°C, 25°C (light conditions) and 25°C (dark conditions).

Storage interval	Parameters / storage conditions (R, L, D)								
	Cyd-3-gal			Cyd-3-glu			Cyd-3-ara		
	4°C	25°C (light conditions)	25°C (dark conditions)	4°C	25°C (light conditions)	25°C (dark conditions)	4°C	25°C (light conditions)	25°C (dark conditions)
15	5.63*	9.63***	4.23*	2.25*	8.95**	3.15*	2.88*	8.82**	3.41*
30	3.40*	28.25***	8.44**	3.01*	27.98***	7.65**	4.63*	42.03***	9.88***
60	5.92*	75.17***	12.05***	3.53*	113.92***	17.09***	9.50**	92.79***	15.57***
180	5.80*	2321.37***	85.42***	7.16**	1371.55***	102.89***	6.41**	1746.27***	146.06***

Asterisks signify the levels of statistical significance of differences compared to initial values: * $p < 0.05$; ** $p < 0.001$; *** $p < 0.0001$

Table S2. The Student's *t*-test results (*t*-values) for statistical comparison of individual anthocyanins from each jam regardless storage conditions. (Red values reveal significant differences, $p < 0.05$)

Anthocyanin		Jam 1 (white sugar)	Jam 2 (fructose)	Jam 3 (erythritol)	Jam 4 (brown sugar)	Jam 5 (coconut sugar)	Jam 6 (stevia)	Jam 7 (saccharine)
**Cyd-3-gal**	Jam1	0.00	0.08	1.93	1.59	4.08(p=0.0018)	0.06	1.68
	Jam2		0.00	1.89	1.79	4.18(p=0.0015)	0.01	1.97
	Jam3			0.00	-0.06	3.11(p=0.0099)	-2.30(p=0.0421)	0.45
	Jam4				0.00	3.86(p=0.0027)	-1.67	0.88
	Jam5					0.00	-3.56(p=0.0045)	-2.89(p=0.0147)
	Jam6						0.00	4.76(p=0.0006)
	Jam7							0.00
**Cyd-3-glu**	Jam1	0.00	1.06	1.87	1.99	4.71(p=0.0006)	1.35	2.87(p=0.0151)
	Jam2		0.00	1.01	1.62	4.50(p=0.0009)	0.75	3.00(p=0.0120)
	Jam3			0.00	1.18	4.19(p=0.0015)	-0.39	2.24(p=0.0470)
	Jam4				0.00	4.69(p=0.0007)	-1.39	1.58
	Jam5					0.00	-4.37(p=0.0011)	-3.47(p=0.0054)
	Jam6						0.00	2.51p=(0.0288)
	Jam7							0.00
**Cyd-3-ara**	Jam1	0.00	0.81	1.97	2.19	3.71(p=0.0035)	1.17	1.54
	Jam2		0.00	1.71	1.95	3.43(p=0.0056)	0.83	1.61
	Jam3			0.00	1.30	2.66(p=0.0221)	-1.12	0.33
	Jam4				0.00	2.62(p=0.0239)	-1.65	-0.45
	Jam5					0.00	-3.30(p=0.0072)	-2.45(p=0.0322
	Jam6						0.00	1.54
	Jam7							0.00

Table S3. The value of percentage enzyme inhibition of lingonberry jams. The value are presented as mean of three determination ±SD.

	**Jam 1 – sucrose**	**Jam 2 – fructose**	**Jam 3 – erythritol**	**Jam 4 – brown sugar**	**Jam 5 - coconut sugar**	**Jam 6 - stevia**	**Jam 7 – saccharine**	**Unsweetened jam**
**% Inhibition degree**	17.7±7.0	44.1±2.0	35.6±3.5	13.4±3.5	35.8±2.0	40.4±4.2	25.1±1.9	57.0±1.4

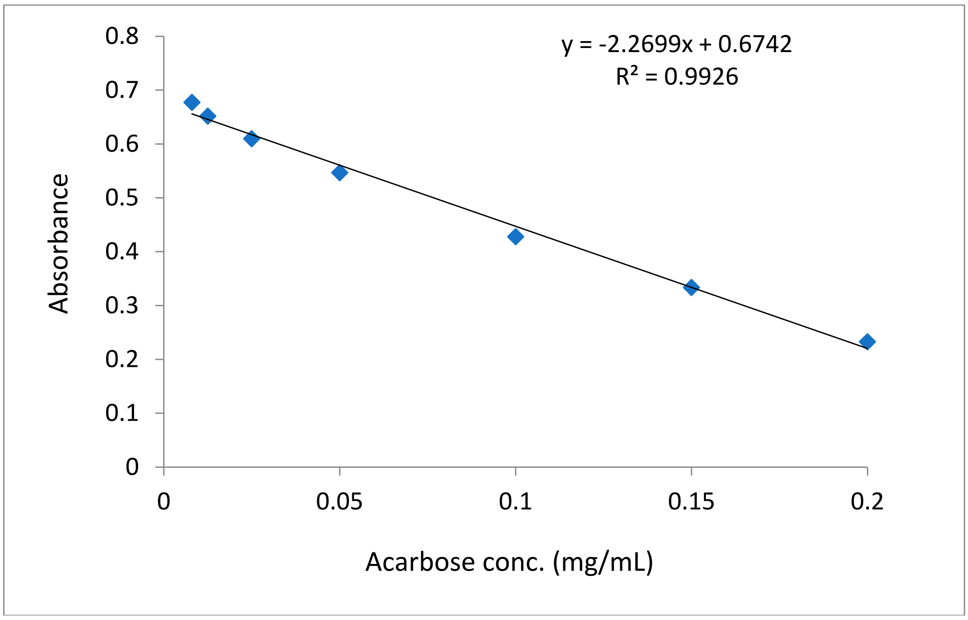

Figure S1. The standard curve of acarbose inhibition effects on the α-glucosidase.

Table S4. The concentration (μM) of each anthocyanin in the extracts of jams administered to rats. The value are presented as mean of three determination ±SD.

**Jam**	**Cy-3-gal**	**Cy-3-glu**	**Cy-3-ara**
Jam 1 (white sugar)	356.8±3.4	29.42±0.88	51.84±0.28
Jam 2 (fructose)	350.2±4.2	26.44±0.28	49.00±0.28
Jam 3 (erythritol)	273.4±3.1	21.4±0.34	40.80±0.57
Jam 4 (brown sugar)	336.2±6.5	24.98±0.20	46.80±0.57
Jam 5 (coconut sugar)	184.4±3.4	12.9±0.20	25.4±0.28
Jam 6 (stevia)	305.6±2.8	22.9±0.20	42.00±0.57
Jam 7 (saccharine)	301.0±1.4	22.04±0.34	43.40±0.28

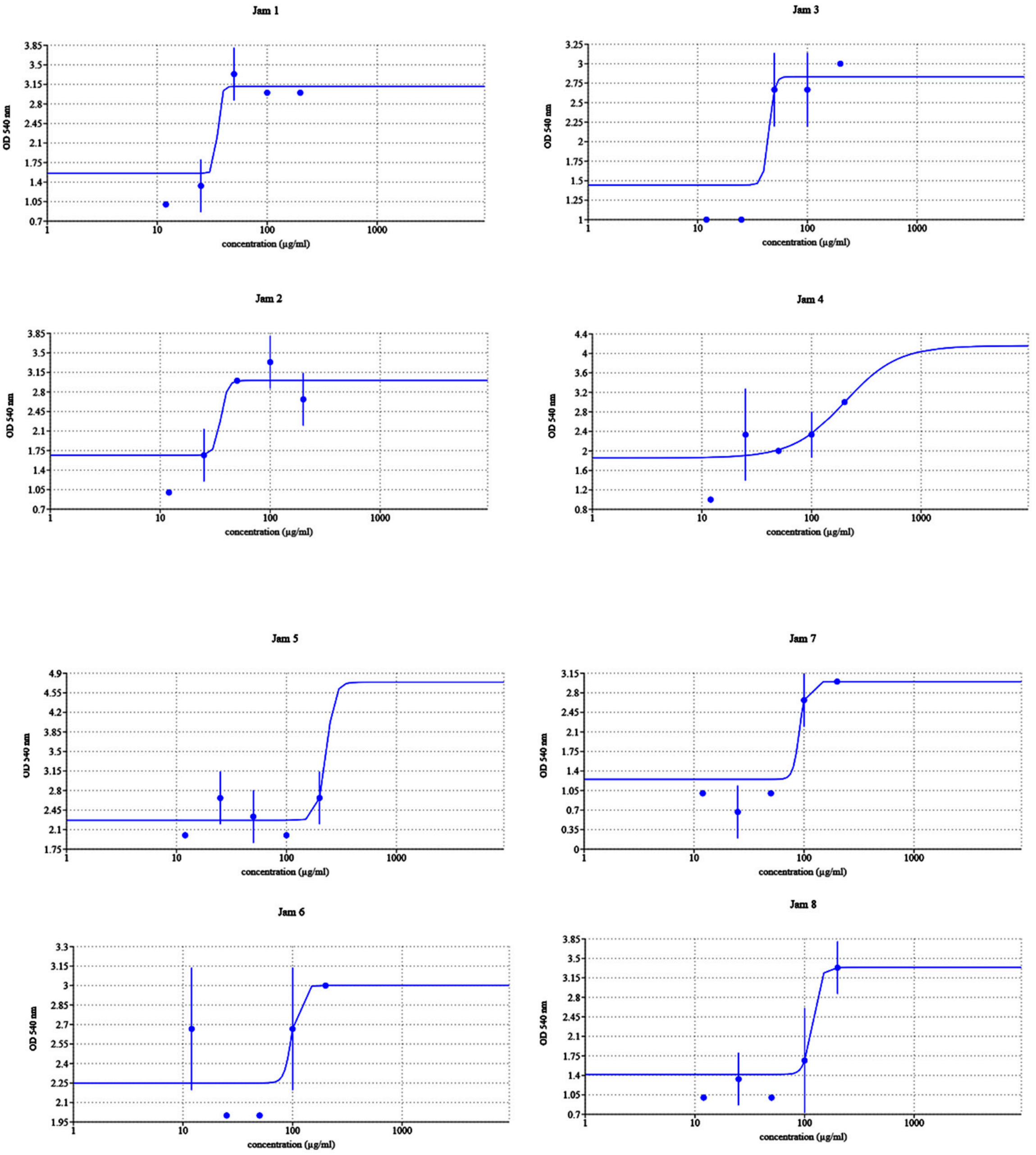

Figure S2. IC50 calculation plots in CACO2 cell line treated with the lingonberry extract jams with different sweeteners. Jam 1=white sugar, jam2 = fructose, jam 3= erythritol, jam 4=brown sugar, jam 5= coconut sugar, jam 6= stevia, jam 7= saccharine, jam 8= unsweetened. Data are presented as OD540 readings, media ±SD, n=3.
